# MIS lateral ACR for spinal deformity correction: technique and complication avoidance

**DOI:** 10.3171/2020.1.FocusVid.19715

**Published:** 2020-01-01

**Authors:** Ifije Ohiorhenuan, Vedat Deviren, Juan S. Uribe

**Affiliations:** 1 Department of Neurological Surgery, Barrow Neurological Institute, St. Joseph’s Hospital and Medical Center, Phoenix, Arizona; and; 2 Department of Orthopaedic Surgery, University of California, San Francisco, California

**Keywords:** anterior column resection, transpsoas approach, deformity correction, video

## Abstract

Deformity correction using minimally invasive surgical (MIS) techniques can be challenging. Here the authors present a case in which an anterior column resection was performed using an MIS lateral approach to restore lumbar lordosis and improve sagittal balance. The authors demonstrate the technique and discuss potential complications and how they may be avoided.

The video can be found here: https://youtu.be/XjOdDeKrKEE.

## Transcript

This video presents the minimally invasive lateral approach for anterior column release. This is a powerful minimally invasive surgical technique for deformity correction, capable of restoring lumbar lordosis and improving sagittal balance. The procedure involves a lateral approach to the lumbar spine (via a transpoas approach), followed by discectomy and release of the ALL with placement of a hyperlordotic implant.

The image in the middle shows a cadaveric specimen of the lumbar spine with half of the ALL sectioned. The image on the right shows a typical hyperlordotic implant. A key feature of this implant are the tabs with screws which secure the implant to the adjacent vertebral bodies. This is critical to prevent anterior migration of the implant once the ALL has been cut.

The ACR offers several advantages. It provides an effective MIS way to provide lordosis. It avoids creating osteotomies. It can be combined with traditional posterior column osteotomies. It provides anterior column support and indirect decompression. It avoids manipulation of neural structures and offers all the advantages of anterior approaches to the spine.

In 2018 a classification for anterior column realignment was published (Uribe et al., 2018). A six-part grading scheme was devised. Grade A described ALL release and hyperlordotic cage placement without posterior element involvement. Grade 1 described ALL release, hyperlordotic cage placement, and inferior facetectomy. Grade 2 described ALL release, hyperlordotic cage placement, and posterior column osteotomies. Grades 3 and 4 described ALL release, hyperlordotic cage placement, and three-column osteotomies. Grades 3 and 4 were differentiated from each other based on the location of the implant relative to the pedicle subtraction osteotomy. Grade 5 described a vertebrectomy with ALL release and corpectomy cage placement.

### 1:57 Case illustration

As an illustrative case, we present this 61-year-old female patient with a history of progressive low-back and leg pain. She had an ODI of 52%. She had failed conservative management and had a normal neurologic exam.

Shown here are her preoperative standing scoliosis films. Her coronal balance was 1.2 cm and her lumbar curve was 31°. Her sagittal balance was negative 0.5 cm. Pelvic incidence of 44° and lumbar lordosis of 17° with a PI-LL mismatch of 27°.

Surgical plan was for a two-stage procedure. Stage 1: T11 to ilium percutaneous segmental fixation; L2/3 and L3/4 complete facetectomies and decompressive foraminotomies; L5–S1 ALIF. Stage 2: L1–2, L2–3, L3–4, and L4–5 LLIF; L2–3 and L4–5 ACR.

### 2:53 Patient positioning

The patient is positioned in a lateral decubitus position with bumps under the axilla and iliac crest. The patient is taped into position such that a true lateral and A/P image can be obtained with fluoroscopy. The table is broken to provide a surgical corridor between the ribs and the iliac crest.

An approximately 4-cm incision is marked over the targeted disc space.

### 3:15 Incision

The incision is made and the external oblique fascia is opened using monopolar cautery.

Blunt dissection through the external and internal oblique muscles is performed. The dissection continues through the transversalis fascia and muscle until the retroperitoneal fat is encountered.

Blunt finger dissection is used to sweep the retroperitoneal contents anteriorly.

The psoas muscle is identified, and a dilator is placed through the muscle. Triggered directional EMG is used to determine the relationship between the dilator and the lumbar plexus.

This clip shows the appropriate dilator position anterior to the lumbar plexus. A higher current threshold is needed to trigger EMG activity when the probe is pointed anteriorly compared to when the probe is pointed posteriorly.

Next, sequential dilation through the psoas is performed with directional triggered EMG used with each dilator.

The retractor is then secured in place and opened.

An annulotomy is made and the disc is removed in standard fashion. It is important to release the contralateral annulus to facilitate placement of cage that spans the full width of the endplate.

The anterior longitudinal ligament is now carefully dissected. Very little resistance should be encountered. Fluoroscopy should be frequently used. It is important that the dissector does not pass the distal pedicle.

This shows how the blade slides within a groove of the ALL dissector.

### 5:00 ACR

Bipolar cautery is used to cauterize the ALL. The ALL is then sectioned with an annulotomy blade. Cuts are made from an anterior to posterior direction. Only the first two-thirds of the ligament should be cut.

A distractor is then used to open up the disc space and fracture the remaining ligament.

Next the implant is advanced into position and secured in place.

### 5:48 Retractor removal

Hemostasis is obtained and the retractor is removed.

Shown here are scout images from CT scans after her stage 1 and stage 2 procedures. Here we can see a comparison of pre- and postop imaging. As can be seen, the ACR resulted in a significant increase in lumbar lordosis and restoration of a physiologic sagittal balance.

### 6:17 Postop course

Postoperatively the patient did well; she was discharged to rehab on postop day 3. At 1-year follow-up she had no back pain and was ambulating increasing distances. ODI at the time was 12%.

### 6:31 Potential ACR complications

There are a number of potential complications related to the ACR. These include vascular and nerve injuries, as well as injuries to the visceral organs. In addition, there can be hardware-related complications.

Complications can generally be avoided by using careful surgical technique and understanding the regional anatomy.

For instance, careful muscle-splitting technique should be used to avoid injuring superficial nerves, which could lead to an abdominal pseudohernia.

Careful technique can also avoid complications such as contralateral psoas hematoma, shown on the left, and violation of endplates and oversizing of the graft, which could lead to subsidence, shown on the right.

An understanding of the regional anatomy is needed to help minimize complications associated with the ACR. The lumbar plexus is intimately related to the psoas muscle.

Shown in this cadaveric dissection are the lumbar nerves exiting from the foramen and forming the lumbar plexus directly underneath the psoas muscle.

This is an MRI from a patient who had a postoperative lumbar plexus neuropraxia caused by stretch injury of the plexus over the graft.

To avoid injury to the lumbar plexus when using a transpsoas approach, triggered directional EMG is critical. This picture shows the appropriate position of a dilator anterior to the lumbar plexus with typical triggered EMG currents shown. This picture shows an incorrect position for the dilator posterior to the lumbar plexus. Again, expected triggered EMG currents are shown.

### 8:22 Incorrect dilator position

This intraoperative clip shows that it is possible to have the dilator positioned incorrectly. Here, small triggered EMG thresholds are observed anteriorly, indicating that the dilator is posterior to the plexus.

It is also important to pay close attention to the vascular anatomy. This clip shows an MRI of a patient whose right iliac vein bifurcates and runs almost underneath the psoas at the L4–5 level. A transpsoas approach from the right side is contraindicated.

### 9:05 Vascular injury

Nevertheless, even with careful surgical technique and an understanding of the vascular anatomy. Vascular injuries can occur during an ACR. In this case, a segmental artery was likely injured.

Principles of vascular repair during an ACR consist of compression, and application of topical hemostatic agents such as FLOSEAL or SURGIFLO. If tear in the vasculature can be visualized, it may be possible to repair it using vascular clips. Ultimately, repair of the injury may require endovascular stenting or open vascular control.
